# An Oligomannuronic Acid-Sialic Acid Conjugate Capable of Inhibiting Aβ42 Aggregation and Alleviating the Inflammatory Response of BV-2 Microglia

**DOI:** 10.3390/ijms222212338

**Published:** 2021-11-15

**Authors:** Jianrong Wu, Miaosen Wu, Hongtao Zhang, Xiaobei Zhan, Nian Wu

**Affiliations:** 1 Key Laboratory of Carbohydrate Chemistry and Biotechnology, Ministry of Education, School of Biotechnology, Jiangnan University, Wuxi 214122, China; wu1094482998@foxmail.com (M.W.); htzhang@jiangnan.edu.cn (H.Z.); xbzhan@jiangnan.edu.cn (X.Z.); 2 Division of Histology and Embryology, International Joint Laboratory for Embryonic Development and Prenatal Medicine, Medical College, Jinan University, Guangzhou 510632, China; nian5999@sina.com

**Keywords:** oligomannuronic acid, sialic acid, Alzheimer’s disease, β-amyloid, microglia

## Abstract

Oligomannuronic acid (MOS) from seaweed has antioxidant and anti-inflammatory activities. In this study, MOS was activated at the terminal to obtain three different graft complexes modified with sialic acid moiety (MOS-Sia). The results show that MOS-Sia addition can reduce the β-structure formation of Aβ42, and the binding effect of MOS-Sia3 is more obvious. MOS-Sia conjugates also have a better complexing effect with Ca^2+^ while reducing the formation of Aβ42 oligomers in solutions. MOS-Sia3 (25–50 μg/mL) can effectively inhibit the activation state of BV-2 cells stimulated by Aβ42, whereas a higher dose of MOS-Sia3 (>50 μg/mL) can inhibit the proliferation of BV-2 cells to a certain extent. A lower dose of MOS-Sia3 can also inhibit the expression of IL-1β, IL-6, TNF-α, and other proinflammatory factors in BV-2 cells induced by Aβ42 activation. In the future, the MOS-Sia3 conjugate can be used to treat Alzheimer’s disease.

## 1. Introduction

Alzheimer’s disease (AD) is a common progressive neurodegenerative disease that is clinically characterized by cognitive impairments. According to data released by the International Alzheimer’s Association, one patient with dementia occurs worldwide every 3.2 s [1]. The pathogenesis of AD is complex and inconclusive. The pathological features of AD patients primarily show senile plaques formed by the aggregation of β-amyloid and intracellular neurofibrillary tangles caused by the phosphorylation of tau protein. However, all of the already established treatments that are approved today try to counterbalance the neurotransmitter imbalance of the disease. For example, acetylocholinesterase inhibitors include Donepezil, Galantamine, and Rivastigmine. Another therapeutic agent approved for moderate to severe AD is the noncompetitive N-methyld-aspartate (NMDA) receptor antagonist Memantine [2]. No new drug has been approved by FDA for AD since 2003 despite many long and expensive trials, and more than 200 research projects in the last decade have failed or have been abandoned [3]. The recent approval request to the FDA for the monoclonal antibody Aducanumab (Biogen) is also controversial [4]. Following these controversies, the FDA did not grant full approval of the Aducanumab medication.

Sialic acid is a type of 9-C ketonic acid extensively present in the sugar-chain terminal of cellular glycoproteins, glycolipids, and small RNA [5]. Sialic acid plays an important role in neural development, cell recognition, tumor migration, and other biological processes. Numerous sialylated glycans exist in human brain tissues. In neonatal brains, sialic acid primarily exists in the form of polysialic acid (PSA), in neural-cell adhesion molecule, and PSA vanishes after adulthood. Given the strong negativity and non-immunogenicity of PSA, several groups have attempted to construct a sialic acid complex for AD treatment. For instance, a polyamidoamine dendrimer grafted with sialic acid was synthesized to mimic the PSA structure in the neonatal brain, and this dendrimer complex could alleviate β-amyloid-induced neurotoxicity [6]. Furthermore, three different sialylated oligosaccharides (DSLNT, LNNT, and 3SL) were photo-crosslinked to obtain PSA analogs. Results show that only the polymer from DSLNT can reduce the neurotoxicity of Aβ, and a higher dose of cross-linked DSLNT can attenuate the toxicity from Aβ [7]. In microglia, Siglec 3 (CD33) expression is found to be positively correlated with Aβ plaque burden, and CD33 inactivation by synthetic analogs of sialic acid could reduces the insoluble Aβ42 levels in APPSwe/PS1ΔE9 mouse brain [8]. A novel nanoparticle embellished with oligo PSA (2–3 DP) could reportedly interact with Siglec-E and inhibit the activation of microglia and inflammatory response [9]. These findings demonstrate that sialic acid containing polymer is a potential PSA analog for attenuating Aβ toxicity relative to the human brain. Given the instability of naturally occurring PSA, a novel structural design of PSA analog is urgent.

Oligomannuronic acid (MOS), a β-D-mannuronic acid polymer linked by β-(1,4) glycosidic bonds, is prepared from the degradation and separation of alginate from seaweed. MOS shows high bioactivity in immune regulation, as well as antitumor, antioxidation, and anti-inflammatory effects [10,11]. MOS can also increase SOD in rat brain tissues, thereby inhibiting brain cell damage caused by oxidative stress, protecting neurons and improving rat cognitive ability [12]. Moreover, MOS can activate the neurotrophic factors of glial cells in a clinical trial, thereby improving neuroprotective function [13]. MOS has also been tested in the prevention and treatment of neurodegenerative diseases. One of its derivatives, GV-971 (an oxidation product at the reducing terminal of MOS), shows an important breakthrough in AD treatment and is approved by CFDA as a cure for patients with mild to moderate Alzheimer’s disease [14]. GV-971 is found to remodel gut microbiota, reduce the accumulation of peripheral metabolites phenylalanine/isoleucine, and reduce neuroinflammation in the brain, resulting in improved cognitive impairment. However, the body adsorption rate of GV-971 through oral administration (900 mg ever day) is very low, and only six-thousandth of it enters the brain.

Reducing Aβ-plaque burden is one strategy for treating AD. Several anti-amyloid aggregation agents such as the oral agent scyllo-inositol and the peptidomimetics KLVFF were developed [15,16]. In addition, abnormal accumulation or dyshomeostasis of metal ions such as iron, copper, and zinc has been associated with the pathophysiology of AD. Some metal chelating agents, such as Deferiprone and PBT2 (a metal protein-attenuating compound), were synthesized and studied in phase 2 trials [17,18]. In the current study, based on the role of sialic acid in biological recognition and the excellent biocompatibility of mannuronic acid, an electronegative oligo mannuronic acid-sialic acid conjugate (MOS-Sia) was synthesized by chemical methods to yield an oligo PSA mimic analog. The interactions of MOS-Sia with Aβ42 and metal ions, as well as its effect on reducing the neurotoxicity of Aβ on microglia, were investigated. The use of MOS-Sia is a potential alternative method of treating cognitive impairment.

## 2. Results

### 2.1. Synthesis and Characterization of MOS-Sia

**Scheme 1:**
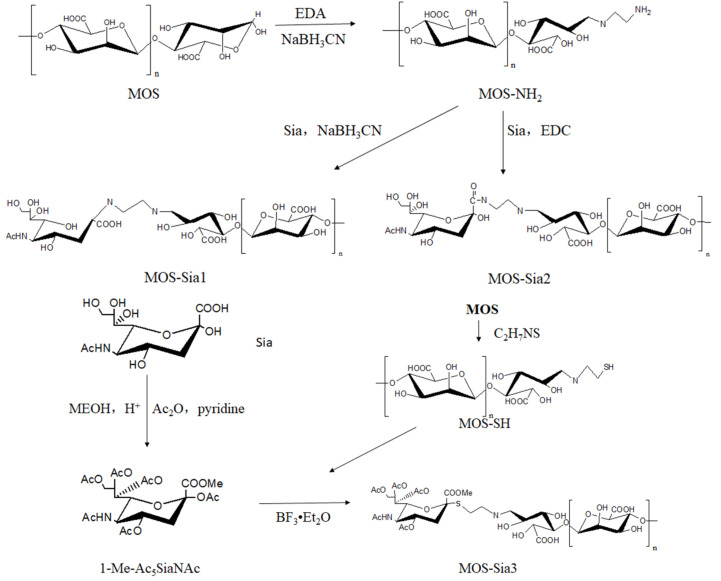
Preparation of MOS-Sia by reductive amination, amidation, and thiol condensation.

MOS-Sia of different structures was synthesized according to Scheme 1. An amino group was introduced into the reducing terminal of MOS (3–5 DP) to produce MOS-NH_2_ through reductive amination (100% yield). MOS-NH_2_ was then reacted with N-acetylneuraminic acid directly through reductive amination, and the obtained conjugate was denoted as MOS-Sia1, with a conversion yield of 75.6% (±0.52). In another trial, MOS-NH_2_ was reacted with N-acetylneuraminic acid under the catalysis of EDC through amidation. The resulting product was denoted as MOS-Sia2, with a conversion yield of 82.7% (±0.63). In the process of alcohol precipitation and dialysis for product purification, MOS-Sia1 and MOS-Sia2 were partially degraded, resulting in decreased yield. In addition, N-acetylneuraminic was initially protected by methyl ester acetylation to generate 1-Me-Ac5SiaNAc, with a yield of 75.6% (±0.38). MOS (3–5 DP) was reacted with mercaptoethylamine to introduce sulfhydryl at the reducing end of oligo mannuronic acid to obtain MOS-SH (yield of 92.5% ± 0.35%). Under the catalysis of boron trifluoride ether (BF3•Et2O), 1-Me-Ac5SiaNAc was reacted with MOS-SH to produce MOS-Sia3, with a yield of 89.2% (±0.42).

**Figure 1:**
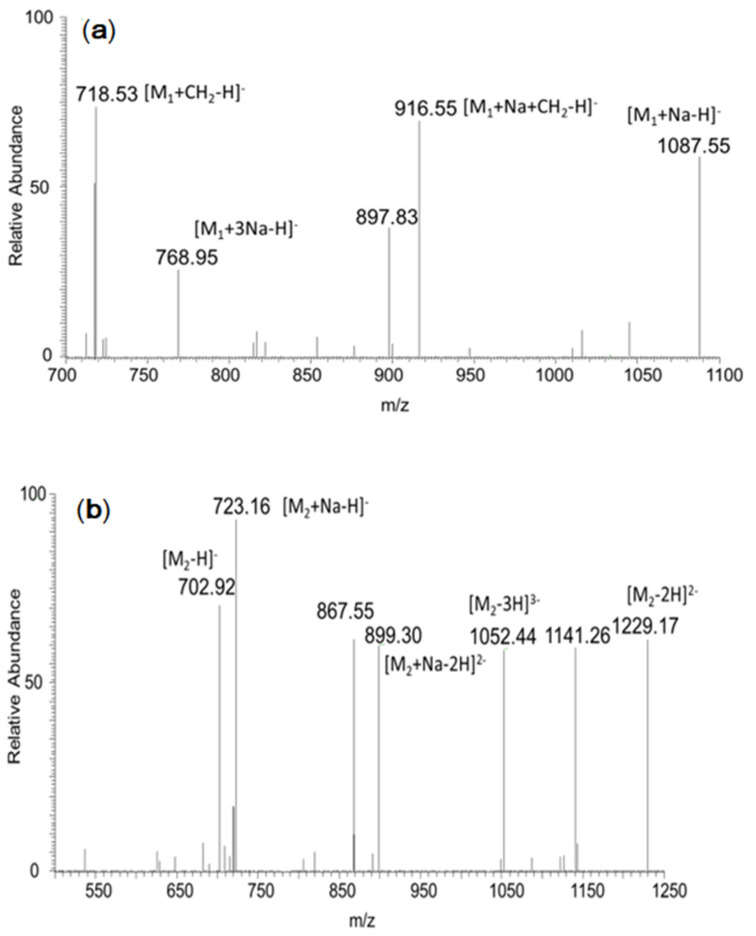

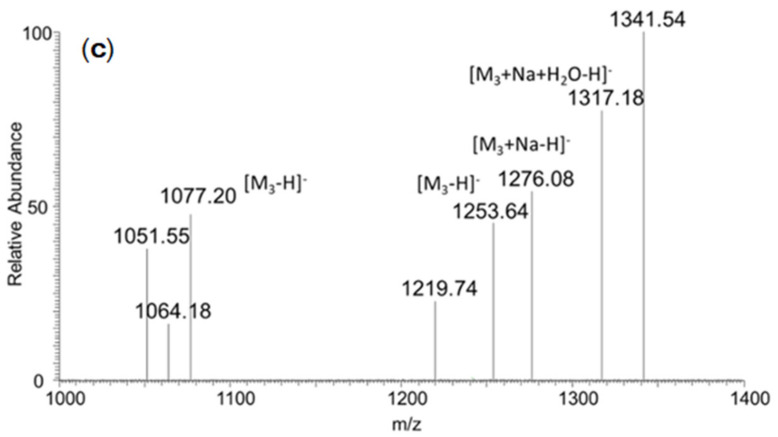
MS spectra of MOS-Sia. (**a**) MOS-Sia1, (**b**) MOS-Sia2, and (**c**) MOS-Sia3.

The MOS-Sia’s were characterized by LC-MS, and the results are shown in Figure 1. Regarding MOS-Sia1, the peaks at *m*/*z* 718.53, 916.55, and 1087.55 corresponded to aminated di-mannuronic acid M_1_+CH_2_-H]^−^, aminated tri-mannuronic acid [M_1_+Na+CH_2_-H]^−^, and aminated tetra-mannuronic acid [M_1_+Na-H]^−^, respectively. With respect to the mass spectrum of MOS-Sia2, *m*/*z* at 702.92, 899.30, 1052.44, and 1229.17 were from amidated di-mannuronic acid [M_2_-H]^−^, amidated tri-mannuronic acid [M_2_+Na −2H]^2−^ and amidated tetra-mannuronic acid [M_2_−3H]^3−^, and amidated penta-mannuronic acid [M_2_ −2H]^2−^, respectively. Mass spectrometry analysis of MOS-Sia3 showed that the peaks at *m*/*z* 1077.2, 1253.64, and 1276.08 corresponded to –SH linked tris-mannuronic acid [M3-H]^−^, –SH linked tetra-mannuronic acid [M3-H]^−^, and –SH linked tetra-mannuronic acid sodium [M3+Na-H]^−^, respectively. Overall, three MOS-Sias were successfully synthesized.

### 2.2. Effect of MOS-Sia on the Sedimentation of Aβ42

**Figure 2:**
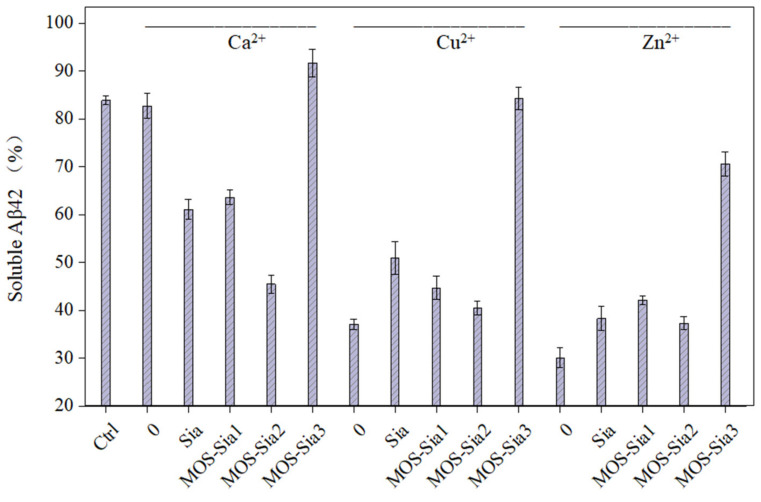
Residual soluble Aβ in supernatant after interaction with MOS-Sia, metal ion, and Aβ42. Note: Molar ratio of Aβ42 to metal ion was set as 1:1, with incubation for 24 h. Data are shown as the mean ± SD of triplicates.

Previous studies have shown that the dynamic imbalance of metal ions such as Ca^2+^, Zn^2+^, and Cu^2+^ could trigger Aβ aggregation in the brain of AD patients, and polysialic acid presence in the brain could reduce cytotoxicity from metal imbalance through chelation [19]. Accordingly, we determined whether MOS-Sia reduced Aβ aggregation and precipitation caused by metal ions, as shown in Figure 2. Ca^2+^ had little effect on the aggregation and precipitation of Aβ42 than compared with Cu^2+^ and Zn^2^. The addition of MOS-Sia1, MOS-Sia2, and Sia reduced soluble Aβ42 content significantly in the Ca^2+^ group. However, the addition of MOS-Sia3 could even increase the soluble Aβ42 content compared with the control. In the presence of Cu^2+^ and Zn^2+^, soluble Aβ was greatly reduced, indicating that these two metal ions were liable to cause Aβ42 to aggregate and precipitate. After adding MOS-Sia or monomer sialic acid, soluble Aβ42 in the supernatant increased in the Cu^2+^/Zn^2^ groups. Among them, the effect of MOS-Sia3 was the most obvious. The electronegativity of and stereoselectivity of MOS-Sia3 may favor its interaction with Aβ42 and inhibit Aβ42 aggregation. The complete structure of sialic acid was destroyed during the synthesis of MOS-Sia1 and MOS-Sia2, proving the importance of the completed terminal sialic acid structure for its interaction with Aβ42 and metal ions. Thus, the electronegative block-sugar complex with a complete sialic acid structure played an important role in stabilizing Aβ42 structure.

### 2.3. Thioflavin T (ThT) Analysis of Interaction between Aβ42 and MOS-Sia

**Figure 3:**
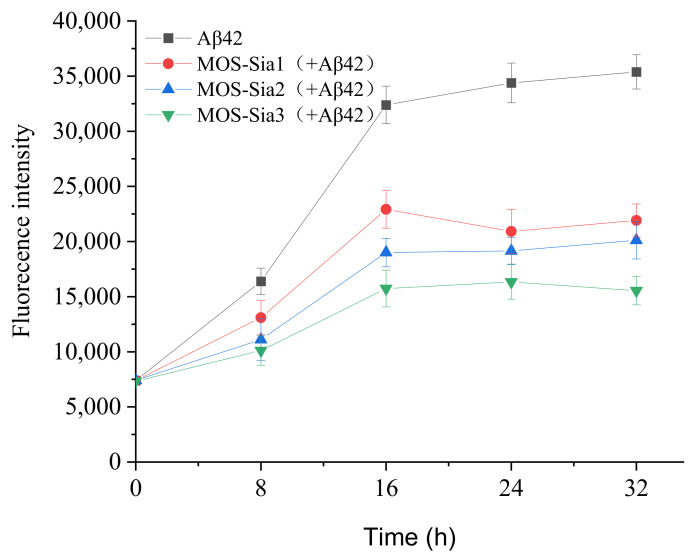
Fluorescence detection of the effect of MOS-Sia on Aβ42 aggregation by ThT staining. Data are shown as the mean ± SD of triplicates.

ThT is a benzoprothiazole dye that specifically binds to Aβ to increase fluorescence with specific excitation wavelength at 450 nm and maximum emission wavelength at 482 nm. Amyloid is rich in β-sheet structure and can specifically bind to ThT, and the presence of ThT does not affect the kinetic process of Aβ aggregation [20]. As shown in Figure 3, all three MOS-Sia had a certain degree of inhibitory effect on Aβ42. After incubation for 32 h, the fluorescence intensity leveled off. MOS-Sia1 and MOS-Sia2 inhibited 50% of ThT fluorescence intensity, whereas MOS-Sia3 inhibited 70% of ThT fluorescence intensity. This finding proved decreased fibril formation from Aβ42 aggregation in the presence of MOS-Sia3.

### 2.4. Circular Dichroism (CD) Analysis of Interaction between Aβ42 and MOS-Sia3

**Figure 4:**
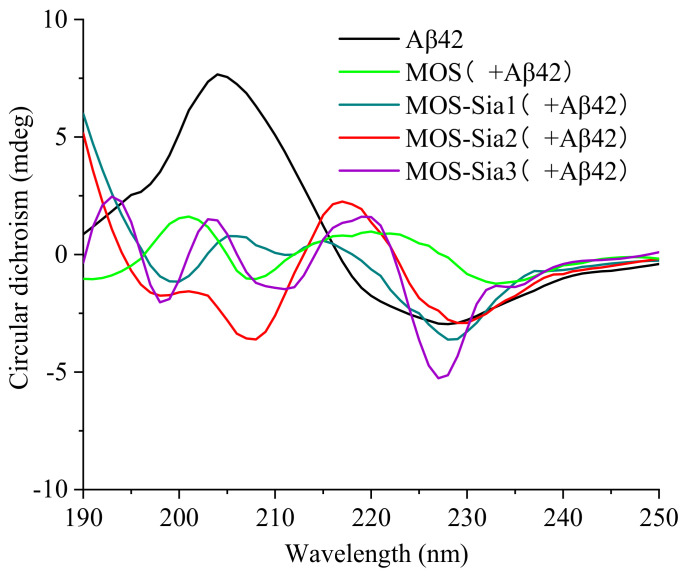
CD analysis of the interaction between MOS-Sia and Aβ42.

The structural change of Aβ42 after interaction with MOS-Sia3 was assayed by CD, and the results are shown in Figure 4. A broad and weak negative peak at 219–228 nm and a positive polarization peak at 200–208 nm were observed for Aβ42 after incubation for 24 h, indicating dominant β sheets and fibril formation with increased molecular weight. After mixing with MOS, the negative peaks at 206–208 nm and the positive peaks at 200–202 and 215–223 nm showed that irregular curls and β-turns dominated. When Aβ42 was incubated with MOS-Sia3, the absorption at around 192 nm increased, and the positive peak absorption at 206 nm was greatly reduced, indicating that MOS-Sia3 addition slowed down the formation of β-sheet structure and reduced the transformation of Aβ42 to β-sheet structure. Moreover, double negative peaks at 207–210 and 226–228 nm, weak positive peaks at 192–194 nm, and wider positive peaks at 223 nm were observed, indicating a typical structure of α-helix with irregular coils. Consequently, MOS-Sia3 showed better binding effects than MOS.

### 2.5. Effect of MOS-Sia3 on the Activity of BV-2 Cells Treated with Aβ42

**Figure 5:**
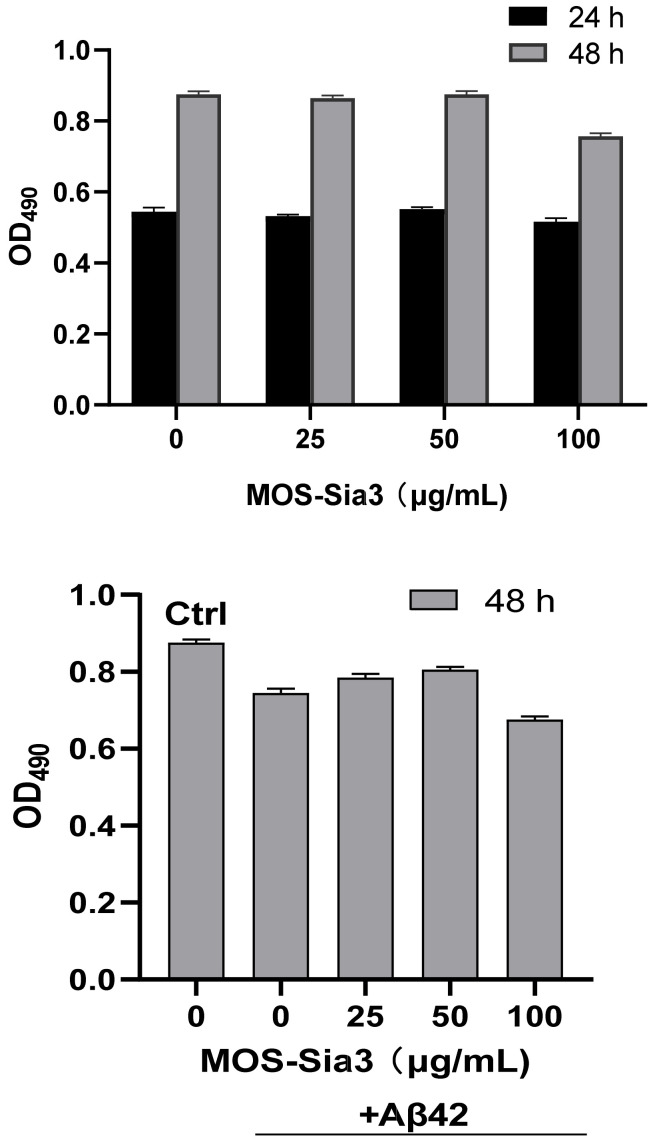
Effect of MOS-Sia3 on the cell growth of BV-2. Data are shown as the mean ± SD of triplicates.

**Figure 6:**
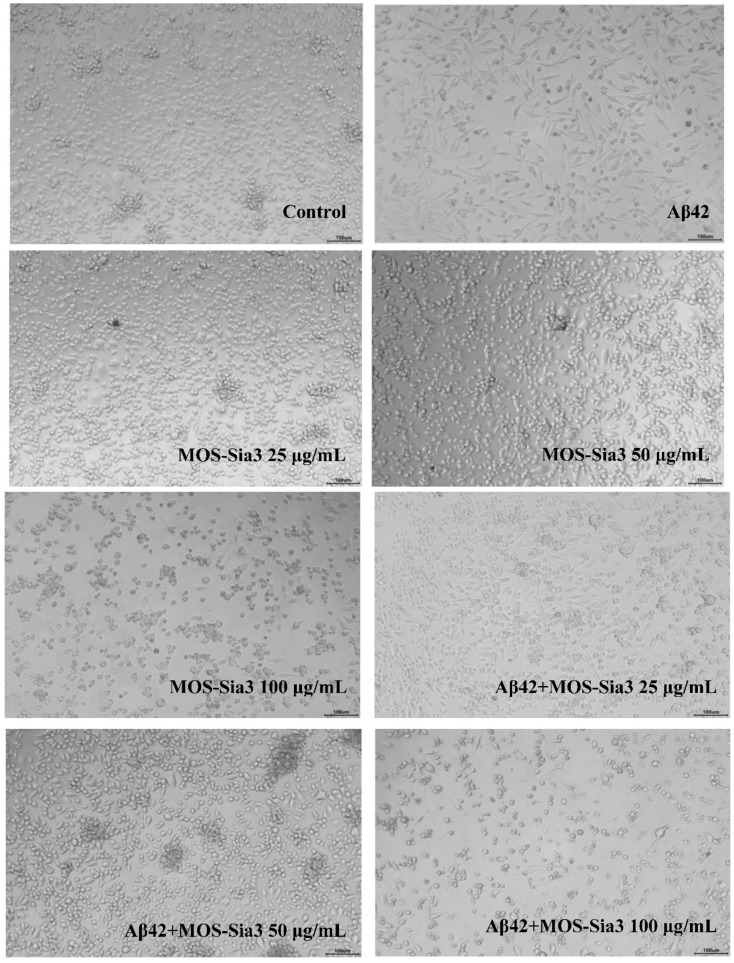
Morphological observation of BV-2 cells after administration with MOS-Sia3 and Aβ42.

Microglia plays a very important role in AD pathogenesis in which Aβ stimulates the activation of microglia to initiate the phagocytosis of Aβ for clearance. Regulating microglia activation is an important target for developing potential therapeutics for AD. The effect of MOS-Sia3 on the cell viability of BV-2 microglia was assayed by the MTT method, and the results are shown in Figure 5. Compared with the control group, the cell-survival rate reached 98.8%, 100.1%, and 86.4% after 48 h of treatment with different concentrations of MOS-Sia3. At a lower concentrations of MOS-Sia3 (25–50 μg/mL), BV-2 cell survival rate remained unaffected. If MOS-Sia3 > 100 μg/mL, cell survival rate was inhibited. When Aβ42 and MOS-Sia3 were used to treat BV-2 cells together, the cell viability of 25 and 50 μg/mL MOS-Sia3 groups slightly increased compared with the group without MOS-Sia3. When MOS-Sia3 > 100 μg/mL, cell viability was also inhibited. The reason may be that the slightly acidic environment caused by the higher concentration of MOS-Sia3 was harmful to BV-2 cells. The morphology of BV-2 cells was observed after treating with MOS-Sia3 and Aβ42 for 48 h, as shown in Figure 6. The normal morphology of BV-2 cells showed round and spindle-shaped adherent morphology. In the presence of Aβ42, BV-2 cells had a small number of adherent cells. High Aβ load is toxic and may cause microglial cell dysfunction due to excessive activation and may increase the risk for dementia [21]. Compared with the Aβ42 model group, a lower concentration of MOS-Sia3 can inhibit the transformation of BV-2 cells into amoebic or long rod-like cells and can also reduce cell apoptosis in the presence of Aβ42. When MOS-Sia3 was >100 μg/mL, MOS-Sia3 had a certain toxic effect on BV-2 and could not inhibit the release pro-inflammatory factors induced by Aβ42 and even increase the secretion of IL-1β and TNF-α. As a result, a high concentration MOS-Sia3 and Aβ42 can inhibit cell-morphology changes, but the proliferation of BV-2 cells was inhibited, which is consistent with the previous MTT assay.

### 2.6. Effect of MOS-Sia3 on the Release of Inflammatory Factors in BV-2 Cells Induced by Aβ42

**Figure 7:**
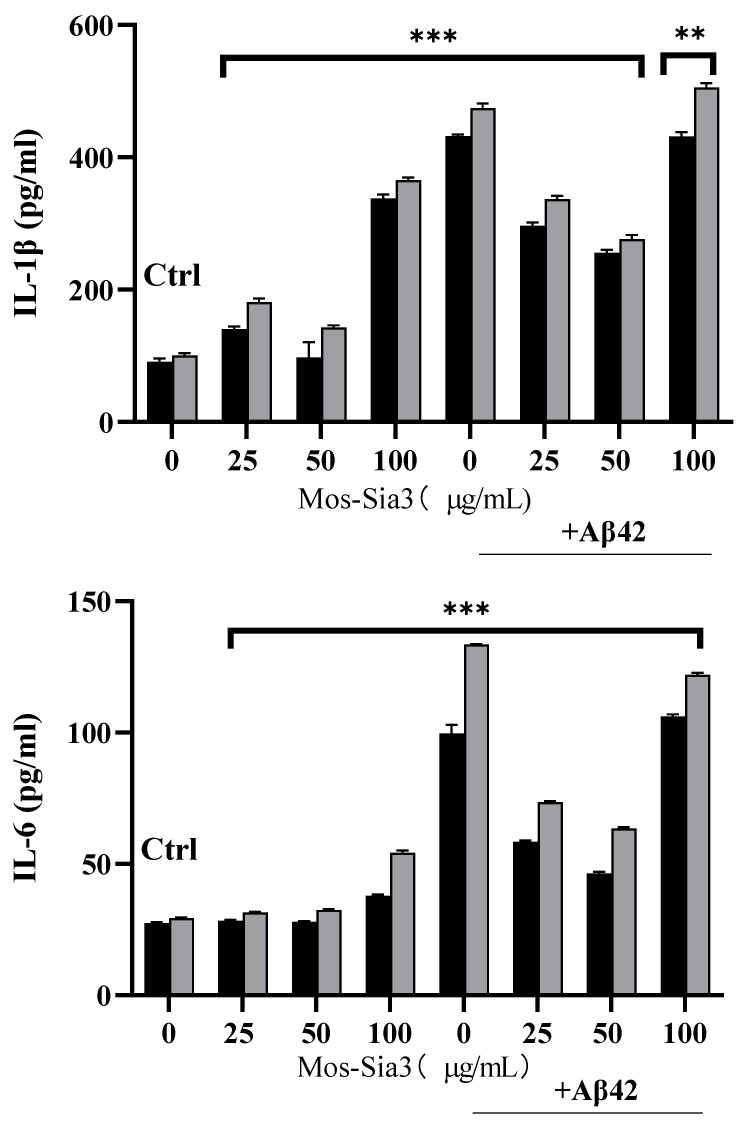

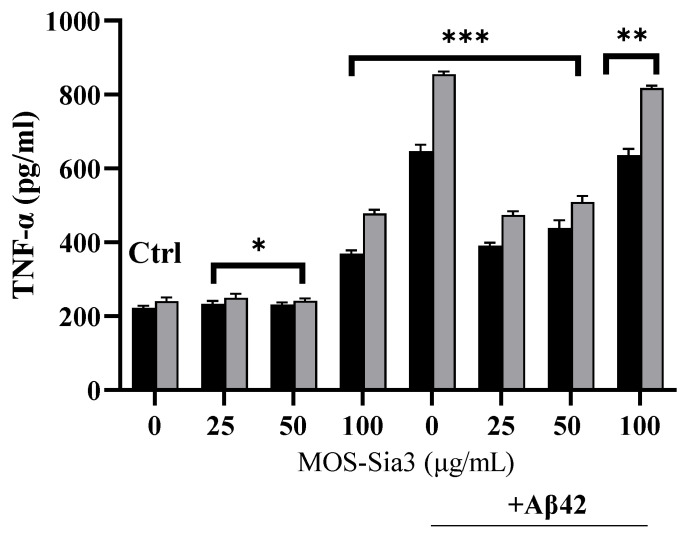
Effects of MOS-Sia3 on cytokine secretion in BV-2 cells induced by Aβ42. Captions: Black bar, 24 h of culture; gray bar: 48 h of culture. Data are shown as the mean ± SD of triplicates with good reproducibility. SD is represented as error bars. Statistically significant differences are indicated: ** for *p* ≤ 0.01, and *** for *p* ≤ 0.001.

Aβ can activate the phagocytic function of microglia. It also promotes the secretion of high levels of pro-inflammatory factors such as IL-1β, IL-6, and TNF-α, resulting in neuron dysfunction or death, thereby causing inflammation. The release of IL-1β, IL-6, and TNF-α in BV-2 cells were measured by ELISA, and the results are shown in Figure 7. Compared with the control group, the level of IL-1β secreted by BV-2 of the Aβ42 model group significantly increased, and secretion reached 474.1 ± 2.1 pg/mL at 48 h. After administration with MOS-Sia3 25 and 50 μg/mL (the molar ratio of MOS-Sia3/Aβ42 was 1:1.13 and 1:2.25, respectively), IL-1β production was significantly inhibited in BV-2 cells, indicating that MOS-Sia3 exerted a certain inhibitory effect on the inflammatory response produced by Aβ42 and can reduce the degree of cell damage caused by Aβ42. The addition of 25–50 μg/mL MOS-Sia3 alone had little effect on IL-1β secretion. However, in the case of 100 μg/mL, the release of IL-1β was close to that of the Aβ42 model group alone. In the presence of 100 μg/mL MOS-Sia3 and Aβ42 (molar ratio of MOS-Sia3/Aβ42 = 1:4.52), IL-1β secretion further increased. With respect to IL-6, its secretion in the Aβ42 model group significantly increased compared with that of the control group, and secretion reached 133.5 ± 1.0 pg/mL at 48 h. In the MOS-Sia3 administration group at 25 and 50 μg/mL, the secretion of IL-6 in BV-2 cells was significantly inhibited, indicating that MOS-Sia3 had a certain inhibitory effect on the inflammatory response induced by Aβ42 and that it can reduce the degree of cell damage caused by Aβ42. The addition of MOS-Sia3 alone had little effect on the release of IL-6 in BV-2. In the presence of 100 μg/mL MOS-Sia3 and Aβ42 (molar ratio of MOS-Sia3/Aβ42 = 1:4.52), the secretion of IL-6 was close to that of the Aβ42 model group. For TNF-α release, the trend was the same as that of IL-1β release. Compared with the blank group, the secretion of TNF-α in the Aβ42 model group significantly increased, and secretion reached 854.2 ± 3.2 pg/mL at 48 h. The addition of MOS-Sia3 at 25 and 50 μg/mL significantly inhibited the secretion of TNF-α in BV-2 cells, whereas a higher titer of 100 μg/mL MOS-Sia3 cannot inhibit IL-1β release.

## 3. Discussion

Sialic acids play various roles in human physiology and pathophysiology. The highest levels of sialic acid are found in the brain, where they are expressed primarily in gangliosides and PSA (primarily in NCAM). PSA is an important regulator of neurogenesis that contributes to neuronal repair and recovery from neurodegeneration such as in AD [22]. However, in aged brains, the capacity of PSA regeneration is significantly reduced [23]. In order to overcome the structural instability of exogenous PSA, an oligosialic acid mimic with a relatively stable structure and negative charge was synthesized by using MOS and sialic acid monomer in this study. MOS-Sia3 can effectively inhibit the activation of BV-2 cells stimulated by Aβ42 within a certain concentration range (<50 μg/mL) and keeps the cells in a normal physiological state. A higher concentration of MOS-Sia3 has the same effect of inhibiting cell-morphology changes, but it can inhibit the proliferation of BV-2 cells to a certain extent. When MOS-Sia3 is >100 μg/mL, it has a certain toxic effect on BV-2 and cannot inhibit the release of pro-inflammatory factors induced by Aβ42, and it can even increase the secretion of IL-1β and TNF-α. BV-2 cells activated by Aβ initially release IL-1β and TNF-α, and then the cascade of TNF-α secretion damages neurons, further resulting in the release of IL-6. Among these pro-inflammatory factors, TNF-α can promote the deposition of Aβ, thereby reducing the ability of BV-2 cells to clear Aβ. As a result, an appropriate dose of MOS-Sia3 was selected in the present study when considering all nervous system cells.

Several functional oligosaccharides have been found to mitigate AD symptoms, especially those from food-derived antioxidant polysaccharides [24]. For instance, the low-molecular-weight heparin can inhibit the transformation of Aβ from random coil to β-sheet, among which the persulfated heparin tetrasaccharide is the best one [25]. A series of heparin oligosaccharide derivatives was designed and synthesized for the inhibition of Aβ40 aggregation through molecular docking and molecular dynamics simulation [26]. Two N-Acetyl chitooligosaccharides (pentasaccharides and tetrasaccharides) were synthesized and could improve the acquisition (learning) and retention (memory) of AD rats [27]. Furthermore, κ-carrageenan oligosaccharides were found to exert a good neuroprotective effect by inhibiting the inflammatory response of microglia [28]. Furthermore, selenized, phosphorylated, and terminal oxidized derivatives of MOS reportedly show antioxidation and anti-neuroinflammation effects [29,30]. Among them, the terminal oxidized derivative, GV-971, has been approved as a treatment for mild to moderate AD patients [14].

Microglial phagocytosis of Aβ is an important function for protecting the brain from pathogenic invasion and for maintaining brain hemostasis. However, the balance between microglial activation and inflammation is disrupted in AD, resulting in chronic neuroinflammation. A variety of Siglecs including CD33, Siglec-11, and Siglec-16 in human microglia can interact with sialic acid to allow microglia to return to an “off” sate [22]. Previous studies have shown that a biomimetic compound containing sialic acid structures similar to those expressed on neuronal membranes can compete and prevent cell–surface Aβ binding and associated cellular damage, thereby reducing the development of Aβ pathology. For instance, a selenium nanoparticle modified with sialic acid and alternative B6 peptide conjugation (B6-Sia-SeNPs) has shown high blood–brain barrier permeability, as well as the abilities to inhibit Aβ fibrillation and reduce Aβ toxicity in a dose-dependent manner in PC12 and bEnd3 cells [31]. Additionally, a sialic-conjugated chitosan has been proven to inhibit Aβ-mediated toxicity in SH-SY5Y cells [32]. Our results proved that MOS-Sia3 can inhibit the aggregation and precipitation of Aβ42, as well as the activation and release of pro-inflammatory factors in microglia within a certain dose. However, further research shall be conducted in AD animal models to test the Aβ-toxicity reduction effect of MOS-Sia3.

## 4. Materials and Methods

### 4.1. Materials

N-Acetylneuraminicacid (Sia) was purchased from Chemstan Biotechnology Co., Ltd. (Wuhan, China). An ultrafiltration centrifuge tube (3 KDa) and dialysis tube were supplied by Solarbio Life Sciences (Beijing, China). Aβ42 was synthesized with NJPeptide (Nanjing, China). DMEM medium, fetal bovine serum, penicillin/streptomycin, and trypsin were supplied by Gibco Life Technologies (Grand Island, NY, USA). Methylthiazolyldiphenyl-tetrazolium bromide (MTT) was supplied by Lablead Biotechnology (Beijing, China). Other reagents were purchased from Sinopharm (Shanghai, China). Polymannuronic acid (PM; 3–5 KDa) was purchased from Qingdao BZ Oligo Biotech Co., Ltd. (Shandong, China)

### 4.2. Preparation of MOS

MOS was prepared by degradation assisted with microwave treatment. In a typical procedure, PM solution (50 mg/mL, pH 7–8) was treated at 120 °C for 20 min (Multiwave PRO, Anton-Paar, Graz, Austria). After decolorization with activated carbon, filtration, precipitation with alcohol, centrifugation, rotary vacuum evaporation, and lyophilization of the hydrolysate, crude MOS were obtained. The crude MOS was redissolved in distilled water, precipitated multiple times with alcohol, and centrifuged, and then the supernatant was lyophilized. The obtained MOS was dissolved in water and subject to isolation with an ultrafiltration centrifuge tube (3 KDa). The filtrate was discarded, and the retentate was collected and lyophilized to obtain the targeted MOS.

### 4.3. Synthesis of MOS-Sia

An appropriate amount of MOS (30 mg/mL) was dissolved in phosphate buffer (pH 7.2). After reaction with ethylenediamine (0.05 mol/L) and sodium cyanoborohydride (0.1 mol/L) at 30 °C for 36 h, the reducing end of MOS was activated to obtain MOS-NH_2_, which was dialyzed with a molecular-weight dialysis bag (500 Da), lyophilized, and stored in a −20 °C refrigerator.

#### 4.3.1. Synthesis of MOS-Sia by Reductive Amination

In order to synthesize MOS-Sia, MOS-NH_2_ (30 mg/mL) was reacted with N-acetylneuraminic acid (0.015 mol/L) and sodium cyanoborohydride (0.15 mol/L) at 25 °C for 36 h (Scheme 1). The reaction solution was precipitated with five times the volume of ethanol, and the precipitate was dialyzed (500 Da) for 48 h in order to remove unreacted chemicals. The obtained product was rotary evaporated, lyophilized, and denoted as MOS-Sia1.

#### 4.3.2. Synthesis of MOS-Sia by Reductive Amidation

In another method for MOS-Sia synthesis, MOS-NH_2_ (30 mg/mL) was reacted with N-acetylneuraminic acid (5 mmol/L) and EDC (10 mmol/L) at 25 °C for 24 h (Scheme 1). The reaction product was precipitated with an appropriate amount of acetone, and the precipitate was dissolved in distilled water again before dialyzing with a molecular-weight dialysis bag (500 Da; Solarbio Life Sciences). The obtained product was rotary evaporated, lyophilized, and denoted as MOS-Sia2.

#### 4.3.3. Synthesis of MOS-Sia by Thiol Condensation

The reducing end of MOS was activated. In a typical procedure, MOS solution (10 mg/mL, dissolved in PBS) was flushed with N_2_ for 10 min and reacted with mercaptoethylamine (2.5 mg/mL) at 25 °C for 36 h. The obtained product was rotary evaporated, dialyzed with a dialysis tube (500 Da, Solarbio Life Sciences), lyophilized, and denoted as MOS-SH.

According to Scheme 1, N-acetylneuraminic acid was protected by methyl esterification and acetylation [33]. The obtained 1-Me-Ac_5_SiaNAc was dissolved in dichloromethane and reacted with MOS-SH under the catalysis of boron trifluoride ether for 10 h in the presence of Amberlite IR120 resin (Merk, Darmstadt, Germany). The reaction product was rotary evaporated, dialyzed with a dialysis tube (1000 Da; Solarbio Life Sciences), lyophilized, and denoted as MOS-Sia3.

### 4.4. Analytical Methods

The modification rate of amino and sulfhydryl groups on oligosaccharides was determined by the trinitrobenzene sulfonic acid method [34] and the 2-nitrobenzoic acid method [35], respectively. MOS and its grafted products were measured by liquid–mass spectrometry (TSQ quantum Ultra EMR, Thermo Fisher Scientific, Waltham, MA, USA) and Fourier infrared spectroscopy (NEXUS 470, Thermo Nicolet, Waltham, MA, USA). Soluble Aβ was assayed using bicinchonininc acid method with a kit (Beyotime Biotechnology, Shanghai, China).

### 4.5. Interaction of MOS-Sia with Aβ42 and Metal Ions

The interaction of MOS-Sia with metal ions and Aβ42 was investigated according to the previous method with minor modification [36]. First, 50 μM Aβ42 solution (dissolved in 5 mM, pH 7.4 PBS) was incubated with MOS or MOS-Sia (0.5 g/L) at 37 °C for 24 h. In another test, 5 mM or 15 mM of CaCl_2_, CuCl_2_, and ZnCl_2_ were mixed with 5 mM of Aβ42, and then the mixtures were incubated with MOS or MOS-Sia (0.5 g/L) at 37 °C for 24 h. All reaction solutions were measured with a circular dichrograph (MOS-450, BioLogic, Auvergne-Rhone-Alpes, Seyssinet-Parise, France) by using PBS as a control.

### 4.6. Th-T Staining Analysis

Aβ42 (10 mM) and MOS-Sia (0.1 g/L) were dissolved in PBS (pH 7.4), incubated at 37 °C, and sampled every 8 h. Then, 180 μL of Th-T (10 μM) was mixed with a 20 μL sample in 96-well plates and determined with a microplate reader (MK3, Thermo Fisher Scientific, Waltham, MA, USA). The excitation and emission wavelengths were 450 and 485 nm, respectively. The average fluorescence intensity of three tests of each sample was calculated.

### 4.7. Effect of MOS-Sia3 on the Cell Viability of BV-2 Cells and Cytokine Release Induced by Aβ42

Culture of BV-2 cells (Langsheng Biotech Inc., Wuxi, China) was digested with 0.25% trypsin and centrifuged (1000 rpm, 5 min). After counting with a hemocytometer, the cell concentration was adjusted and inoculated in a 96-well plate (100 μL, 5000 cells/well). BV-2 cell culture was grouped as follows: (1) control (DMEM only); (2) Aβ42 model group (added with 40 μg/mL Aβ42); (3) low MOS-Sia3 group (added with 25 μg/mL MOS-Sia3); (4) moderate MOS-Sia3 group (added with 50 μg/mL of MOS-Sia3); (5) high MOS- group (added with 100 μg/mL MOS-Sia3); (6) low MOS-Sia3 + Aβ42 (added with 25 μg/mL MOS-Sia3 and 40 μg/mL Aβ42); (7) moderate MOS-Sia3 + Aβ42 (added with 50 μg/mL MOS-Sia3 and 40 μg/mL Aβ42); and (8) high MOS-Sia3 + Aβ42 (added with 100 μg/mL MOS-Sia3 and 40 μg/mL Aβ42).

The cells were cultivated for 24 h with DMEM. Then, the culture medium was decanted, and cells were cultivated with fresh DMEM supplemented with a certain concentration of Aβ42 or Aβ+MOS-Sia3 for another 24 or 48 h. The culture with fresh DMEM served as a control. Afterwards, the culture was replaced with 90 μL of fresh DMEM. Then, 10 μL of MTT was added to each well, and the mixture was incubated for 3 h at 37 °C in a CO_2_ incubator (5%) in darkness. After aspirating the liquid in the wells, 200 μL of DMSO was added, and the mixture was incubated for 10 min at room temperature in a shaker. The OD value was read with a microplate reader at 490 nm (MK3, Thermo Fisher Scientific, Waltham, MA, USA), and the average cytotoxicity was calculated from data of triplicate tests. Microscopy images of BV-2 cell were obtained with an optical microscope (Leica, Magdeburg, Germany). The supernatants of BV-2 cell culture after incubation for 24 or 48 h were collected by centrifugation (10 min, 3000 rpm) and subjected to cytokine assay through ELISA. IL-1β and TNF-α were measured using assay kits (MU30369/MU30030, Bioswamp, Wuhan, China). IL-6 was determined with an IL-6 assay kit (JL20268, Jianglaibio, Shanghai, China).

### 4.8. Statistical Analysis

Results are shown as the mean ± standard deviation. Statistical comparison of means between and among groups was performed by using one-way ANOVA with the Student’s *t* test (*p* ≤ 0.05) and Tukey’s Test using STATISTICA 13 (Dell, Tulsa, OK, USA).

## 5. Conclusions

Under mild conditions, three different MOS-Sia graft conjugates are successfully synthesized by linking with sialic acid monomer. The interaction between MOS-Sia and Aβ42 can reduce the β-structure formations of Aβ42. MOS-Sia conjugates, especially MOS-Sia3, have a better complexing effect with Ca^2+^ while reducing the formation of Aβ42 oligomers in solution. MOS-Sia3 can inhibit 70% of THT fluorescence intensity. A lower titer of MOS-Sia3 (25–50 μg/mL) can effectively inhibit the activation state of BV-2 cells stimulated by Aβ42, whereas a higher dose of MOS-Sia3 (>50 μg/mL) can inhibit the proliferation of BV-2 cells to a certain extent. MOS-Sia3 can also inhibit the the expression of IL-1β, IL-6, TNF-α, and other proinflammatory factors in BV-2 cells induced by Aβ42 activation, thereby reducing the damage and inflammation of BV-2 cells.

## Abbreviations

ThT	Thioflavin T
AD	Alzheimer’s disease
MOS	oligo mannuronic acid
Sia	sialic acid
PSA	polysialic acid
Aβ	Amyloid β
CD	circular dichroism
PBS	phosphate buffered saline
IL-1β	interleukin-1β
IL-6	interleukin-6
TNF-α	tumor necrosis factor-α

## References

[1] Sošić M., Vuletic V., Tomić Z., Bogdanovic N. (2018). Diagnostic and therapeutic approach to a patient with cognitive impairment. Med. Flum..

[2] Cummings J., Lee G., Ritter A., Sabbagh M., Zhong K. (2019). Alzheimer’s disease drug development pipeline: 2019. Alzheimer’s Dement. Transl. Res. Clin. Interv..

[3] Hukins D., MacLeod U., Boland J.W. (2019). Identifying potentially inappropriate prescribing in older people with dementia: A systematic review. Eur. J. Clin. Pharmacol..

[4] Tagliavini F., Tiraboschi P., Federico A. (2021). Alzheimer’s disease: The controversial approval of Aducanumab. Neurol. Sci..

[5] Flynn R.A., Pedram K., Malaker S.A., Batista P.J., Smith B.A., Johnson A.G., George B.M., Majzoub K., Villalta P.W., Carette J.E. (2021). Small RNAs are modified with N-glycans and displayed on the surface of living cells. Cell.

[6] Patel D.A., Henry J.E., Good T.A. (2007). Attenuation of β-amyloid-induced toxicity by sialic-acid-conjugated dendrimers: Role of sialic acid attachment. Brain Res..

[7] Cowan C.B., Coté G.L., Good T.A. (2008). Development of photocrosslinked sialic acid containing polymers for use in amyloid beta toxicity attenuation. Biomaterials.

[8] Griciuc A., Serrano-Pozo A., Parrado A.R., Lesinski A.N., Asselin C.N., Mullin K., Hooli B., Choi S.H., Hyman B.T., Tanzi R.E. (2013). Alzheimer’s Disease Risk Gene CD33 Inhibits Microglial Uptake of Amyloid Beta. Neuron.

[9] Thiesler H., Beimdiek J., Hildebrandt H. (2020). Polysialic acid and Siglec-E orchestrate negative feedback regulation of microglia activation. Cell. Mol. Life Sci..

[10] Hu X., Jiang X., Hwang H., Liu S., Guan H. (2004). Antitumor activities of alginate-derived oligosaccharides and their sulphated substitution derivatives. Eur. J. Phycol..

[11] Yamamoto Y., Kurachi M., Yamaguchi K., Oda T. (2007). Induction of Multiple Cytokine Secretion from RAW264.7 Cells by Alginate Oligosaccharides. Biosci. Biotechnol. Biochem..

[12] Fan Y., Hu J., Li J., Yang Z., Xin X., Wang J., Ding J., Geng M. (2005). Effect of acidic oligosaccharide sugar chain on scopolamine-induced memory impairment in rats and its related mechanisms. Neurosci. Lett..

[13] Wang X.J., Chen X.H., Yang X.Y., Geng M.Y., Wang L.M. (2017). Acidic oligosaccharide sugar chain, a marine-derived oligosaccharide, activates human glial cell line-derived neurotrophic factor signaling. Neurosci. Lett..

[14] Wang X., Sun G., Feng T., Zhang J., Huang X., Wang T., Xie Z., Chu X., Yang J., Wang H. (2019). Sodium oligomannate therapeutically remodels gut microbiota and suppresses gut bacterial amino acids-shaped neuroinflammation to inhibit Alzheimer’s disease progression. Cell Res..

[15] Salloway S., Sperling R., Keren R., Porsteinsson A., Van Dyck C.H., Tariot P.N., Gilman S., Arnold D., Abushakra S., Hernandez C. (2011). A phase 2 randomized trial of ELND005, scyllo-inositol, in mild to moderate Alzheimer disease. Neurology.

[16] Stark T., Lieblein T., Pohland M., Kalden E., Freund P., Zangl R., Grewal R., Heilemann M., Eckert G.P., Morgner N. (2017). Peptidomimetics That Inhibit and Partially Reverse the Aggregation of Aβ1–42. Biochemistry.

[17] Xu P., Zhang M., Sheng R., Ma Y. (2017). Synthesis and biological evaluation of deferiprone-resveratrol hybrids as antioxidants, Aβ 1–42 aggregation inhibitors and metal-chelating agents for Alzheimer’s disease. Eur. J. Med. Chem..

[18] Krishnan H.S., Bernard-Gauthier V., Placzek M.S., Dahl K., Narayanaswami V., Livni E., Chen Z., Yang J., Collier T.L., Ran C. (2018). Metal Protein-Attenuating Compound for PET Neuroimaging: Synthesis and Preclinical Evaluation of [^11^C]PBT2. Mol. Pharm..

[19] Murthy R.V., Bharate P., Gade M., Sangabathuni S., Kikkeri R. (2016). Effect of Transition Metals on Polysialic Acid Structure and Functions. ChemMedChem.

[20] Younan N.D., Viles J.H. (2015). A Comparison of Three Fluorophores for the Detection of Amyloid Fibers and Prefibrillar Oligomeric Assemblies. ThT (Thioflavin T); ANS (1-Anilinonaphthalene-8-sulfonic Acid); and bisANS (4,4′-Dianilino-1,1′-binaphthyl-5,5′-disulfonic Acid). Biochemistry.

[21] Fang M., Zhao Y., Liu X. (2019). High Aβ load may cause microglial cell dysfunction and reduced nuclear repressor element-1 silencing transcription factor (REST) expression which might be ascribed to its degradation by ubiquitination. Ann. Transl. Med..

[22] Rawal P., Zhao L. (2021). Sialometabolism in Brain Health and Alzheimer’s Disease. Front. Neurosci..

[23] Schnaar R.L., Gerardy-Schahn R., Hildebrandt H. (2014). Sialic Acids in the Brain: Gangliosides and Polysialic Acid in Nervous System Development, Stability, Disease, and Regeneration. Physiol. Rev..

[24] Li H., Ding F., Xiao L., Shi R., Wang H., Han W., Huang Z. (2017). Food-Derived Antioxidant Polysaccharides and Their Pharmacological Potential in Neurodegenerative Diseases. Nutrients.

[25] Zhou X., Jin L. (2016). The Structure-Activity Relationship of Glycosaminoglycans and Their Analogues with β-Amyloid Peptide. Protein Pept. Lett..

[26] Paul T., Kelly H., Zuchniarz J., Ahmed T., Prabhakar R. (2016). Design of heparin oligosaccharide based molecules for inhibition of Alzheimer amyloid beta (Aβ40) aggregation. Can. J. Chem..

[27] Jiang Z., Liu G., Yang Y., Shao K., Wang Y., Liu W., Han B. (2019). N-Acetyl chitooligosaccharides attenuate amyloid β-induced damage in animal and cell models of Alzheimer’s disease. Process. Biochem..

[28] Ai L., Chung Y.-C., Lin S.-Y., Jeng K.-C.G., Lai P.F.-H., Xiong Z.-Q., Wang G. (2018). Carrageenan polysaccharides and oligosaccharides with distinct immunomodulatory activities in murine microglia BV-2 cells. Int. J. Biol. Macromol..

[29] Zhu Z., Liu Q., Chen P., Xu X., Ni J., Yang S., Song Y. (2013). Seleno-polymannuronate synthesis and resistance to oxidation and apoptosis in Alzheimer’s disease cells. Chem. J. Chin. Univ..

[30] Li Q., Zeng Y., Wang L., Guan H., Li C., Zhang L. (2016). The heparin-like activities of negatively charged derivatives of low-molecular-weight polymannuronate and polyguluronate. Carbohydr. Polym..

[31] Yin T., Yang L., Liu Y., Zhou X., Sun J., Liu J. (2015). Sialic acid (SA)-modified selenium nanoparticles coated with a high blood–brain barrier permeability peptide-B6 peptide for potential use in Alzheimer’s disease. Acta Biomater..

[32] Dhavale D.D. (2009). Sialic Acid Conjugated Chitosan for the Attenuation of Amyloid-Beta Toxicity. Master’s Thesis.

[33] Li J., Zhang J., Zhang Q., Bai Z., Zhao Q., He D., Wang Z., Chen Y., Liu B. (2018). Syntheses and anti-cancer activity of CO-releasing molecules with targeting galactose receptors. Org. Biomol. Chem..

[34] Hanh V.T., Kobayashi Y., Maebuchi M., Nakamori T., Tanaka M., Matsui T. (2016). Quantitative mass spectrometric analysis of dipeptides in protein hydrolysate by a TNBS derivatization-aided standard addition method. Food Chem..

[35] Ellman G.L., Courtney K.D., Andres V., Featherstone R.M. (1961). A new and rapid colorimetric determination of acetylcholinesterase activity. Biochem. Pharmacol..

[36] Lei Y., Wu M., Wang J., Zhang H., Zhan X., Sun Z., Wu J. (2019). Preparation and property of a biantenna macromolecule based on polysialic acid. Int. J. Biol. Macromol..

